# Structural effects of intraretinal cysts on outer retinal layers in eyes with diabetic macular edema

**DOI:** 10.1186/s40942-024-00605-w

**Published:** 2024-11-08

**Authors:** Micael Valtoni Dantas do Nascimento, Claudio Iovino, Po Hsiang Shawn Yuan, Haaris M. Khan, Leonardo Provetti Cunha, Leandro Cabral Zacharias, Nehemias Lacerda, Eduardo Navajas, Mario L. R. Monteiro, Rony C. Preti

**Affiliations:** 1 Division of Ophthalmology, University of São Paulo Medical School, São Paulo, Brazil; 2 Multidisciplinary Department of Medical, Surgical and Dental Sciences, Eye Clinic, University of Campania Luigi Vanvitelli, Naples, Italy; 3 Faculty of Medicine, University of British Columbia, Vancouver, BC Canada; 4 Department of Ophthalmology, School of Medicine, Federal University of Juiz de Fora, Juiz de Fora, Brazil; 5 PhD in aeronautics, physics and fluids, California Institute of Technology, Pasadena, USA; 6 Division of Ophthalmology, University of British Columbia, Vancouver, Canada

**Keywords:** Cysts, Macular edema, Diabetic retinopathy, Tomography, optical coherence

## Abstract

**Background:**

Diabetic macular edema (DME) is the main cause of visual loss in individuals with diabetic retinopathy (DR). This study aims to investigate the effects of central macular intraretinal cysts on the underlying outer retinal layer (ORL) in patients with diabetic macular edema (DME).

**Methods:**

In this retrospective and cross-sectional study, diabetic patients with or without DR were categorized into three groups: without DME (group 1), with DME but without any cyst featuring a plateau in the lower region (group 2), and patients with cyst featuring an inferior cyst plateau (group 3), defined as a flat conformation at its posterior aspect. Variables such as central macular intraretinal cyst height, inferior cyst plateau, and ORL thickness were measured, and ellipsoid zone (EZ) disruption was assessed via Spectral-domain optical coherence tomography (SD-OCT) and compared between groups. Correlations between OCT-measured variables and best-corrected visual acuity (BCVA) were investigated.

**Results:**

A total of 164 eyes were included: 48 in group 1, 47 in group 2 and 69 in group 3. Compared with Groups 1 and 2, Group 3 presented a greater intraretinal cyst height (*p* < 0.001), a thinner mean ORL beneath the cysts (*p* < 0.0001) and more frequent EZ disruption (*p* < 0.0001), which was associated with lower BCVA values. Cyst height, cyst plateau and ORL thickness were significantly correlated with BCVA (*p* < 0.0001). EZ disruption was associated with the cyst height, the cyst plateau and the underlying ORL thickness. Correlations were observed between cyst height and ORL thickness (*r* = − 0.32, *p* < 0.001), between cyst height and cyst plateau (*r* = 0.60, *p* < 0.001), and between cyst plateau and ORL thickness (*r* = − 0.56, *p* < 0.001). Every increase of 10 μm in plateau width and in cyst height results in reductions of 0.16 μm and 0.29 μm in ORL thickness, respectively, independent of the other parameters. The optimal cutoff point for cyst height that best discriminates plateau formation was determined to be 130.5 μm, with a sensitivity of 89.9% and specificity of 83%.

**Conclusions:**

In patients with DME, large central intraretinal cysts may assume a flat configuration in their lower region, termed a plateau, and are associated with photoreceptor damage due to compression, which can result in visual impairment. These findings can be understood based on modified Hertz’s mechanical contact theory.

## Background

Diabetic macular edema (DME) is considered the main cause of visual loss in individuals with diabetic retinopathy (DR) [1]. Despite the advent of several therapeutic options to treat DME, physicians have yet to devise methodologies to identify patient categories that would optimally respond to some treatments rather than others [2]. 

Recognizing DME retinal biomarkers is pivotal for understanding DME treatment outcomes. Spectral-domain optical coherence tomography (SD–OCT) is a noninvasive and reliable method of depth-resolved, near-histological high-resolution assessment of cross-sectional transverse retinal tissue for the identification of imaging biomarkers [3–6], including central macular thickness (CMT) [3], the integrity of the outer retinal layer (ORL), the presence of subretinal fluid [7], intraretinal foci and disorganization of retinal inner layers (DRIL) [6, 8]. Intraretinal cysts of varying dimensions characterize DME and lead to increased CMT, which is a revered OCT parameter to prognosticate vision amelioration in treated eyes [3]. 

Previous studies have attempted to elucidate why large retinal cysts represent a more serious threat to visual function in patients with DME [4, 9]. Pelosini and colleagues reported a linear correlation between the residual tissue sandwiched between the inner and outer plexiform retinal layers on OCT B-scans and visual acuity [9]. The larger the intraretinal cysts between the two layers, the less tissue was visible on the B-scan sections of OCT. Notably, they hypothesized that this tissue, bridging retinal photoreceptors to the ganglion cells, comprising bipolar cells and Müller cell bodies, is very important for maintaining retinal structure and function [9]. Impairment in retinal function due to bipolar cell dysfunction could disrupt visual function, a situation exacerbated by retinal thickness augmentation leading to axon stretching and eventual rupture. Moreover, Müller cell displacement or damage caused by intraretinal cysts could be detrimental to bipolar axons, thereby precipitating vision loss [9]. However, this field warrants further exploration, given that structural anatomical changes promoted by edema are not strongly correlated with, nor do they consistently mirror, visual dysfunction [3]. Moreover, since intraretinal cysts can exert substantial pressure on the internal retinal layers, potentially leading to significant damage [10], it is possible that they may also exert pressure on the ORL.

To explain this phenomenon, bioengineering principles can be applied. The mechanical stress exerted by the intraretinal cysts on the surrounding retinal structures, including the photoreceptors and the ORL, can be modeled to understand the pressure distribution and tissue deformation. Hertz’s theory of mechanical contact, a subdiscipline of bioengineering, could elucidate the intricate dynamics, tangential and perpendicular forces, and the complexity involved in this process [11]. This theory specifically addresses the localized stress generated by the contact of two spheres under load. Therefore, we can apply it in cases of CME to analyze the contact between one sphere representing the intraretinal cyst and the other representing the ORL, with an assumed infinite radius [12]. This approach can help clarify how the compression caused by cysts on the ORL leads to cellular damage and visual impairment, providing insights for potential therapeutic strategies.

The purpose of this study was to investigate, using different OCT-measured intraretinal cyst parameters, the repercussions of large DME intraretinal cysts on the underlying retinal layers and their correlation with visual function. We also sought to interpret the genesis and secondary complications of DME intraretinal cysts via modified Hertz’s theory of mechanical contact.

## Methods

This retrospective, observational, cross-sectional, and comparative study was conducted in accordance with the principles of the Declaration of Helsinki and received approval from the Ethics Committee for Analysis of Research Projects of the Hospital das Clínicas of the Faculty of Medicine of the University of São Paulo on July 9, 2020 (CAPPesq n° 4,144,555). We secured informed written consent from all participants prior to enrollment. The study included consecutive type 2 diabetic patients with or without DR, and with or without DME, who underwent examinations at the Division of Ophthalmology of the University of São Paulo Medical School between August 2020 and January 2023. Systemic and ophthalmological data were obtained from retrospective analyses of medical records. An independent researcher, who was blinded to other data, examined the OCT images from the device’s database to obtain and measure the structural variables. A third independent researcher also analyzed the OCT images, without having access to any other analysis or data, to enable an analysis of the agreement between the structural variables.

### Inclusion and exclusion criteria

The participants included type 2 diabetic patients with or without DR, with or without DME, without DRIL and with best-corrected visual acuity (BCVA) ≥ 20/200. We excluded individuals with a history of macular grid laser photocoagulation treatment, pars plana vitrectomy, recent (within 3 months) intravitreal anti-VEGF injections, vitreomacular traction, significant media opacity such as cataract or vitreous hemorrhages, or any other ocular disorder that could impede OCT evaluation. We also excluded individuals whose medical records were incomplete.

### Study cohort and group allocation

**Fig. 1:**
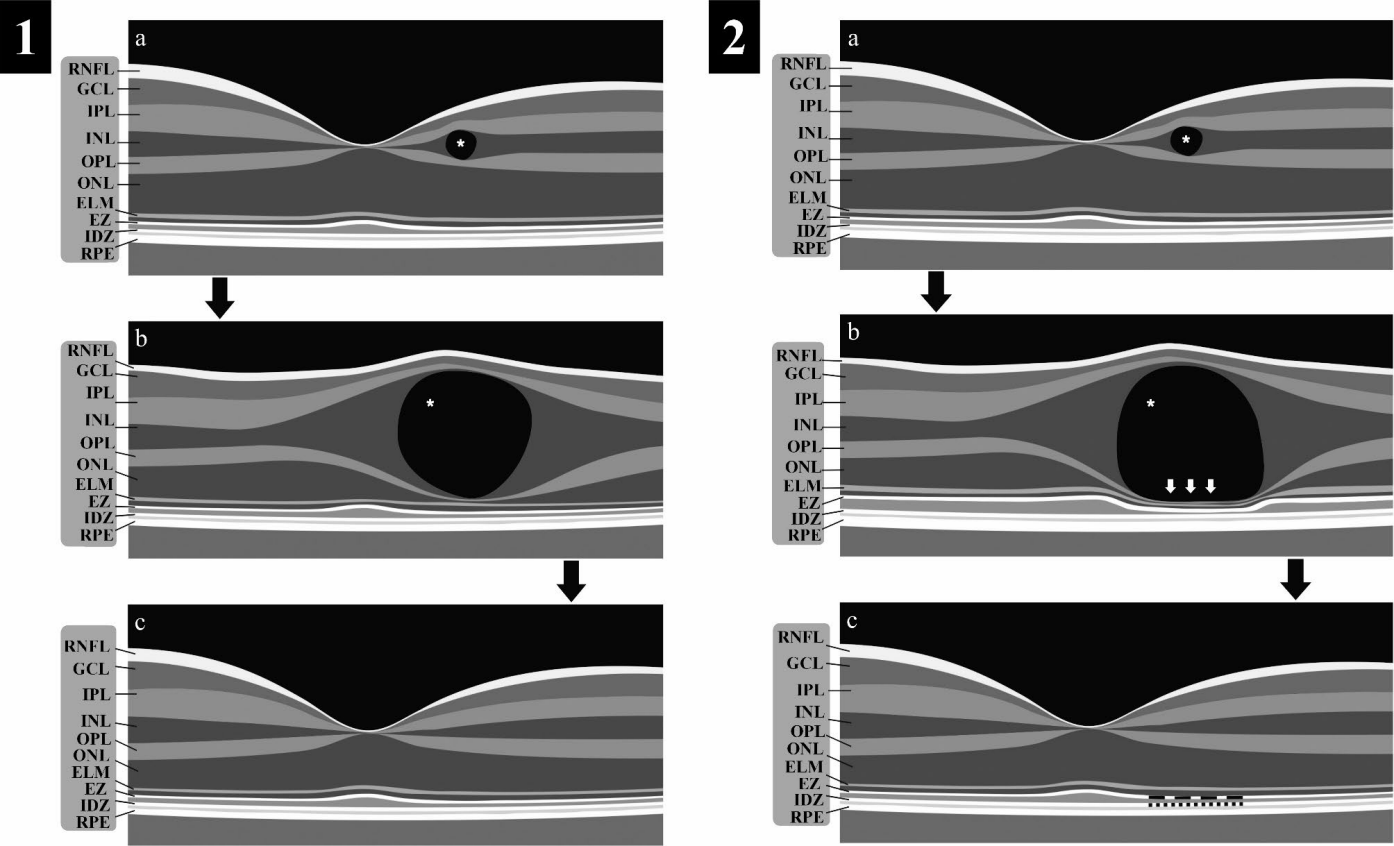
Schematic Figure Detailing the Progression and Resolution of Cysts: **1a** and **2a**: Depiction of the initial appearance of a cyst in the inner nuclear layer (indicated by a white asterisk). **1b**: Illustration of cyst growth, characterized by the absence of a plateau. **1c**: Demonstration of cyst resolution without ensuing damage to the ellipsoid zone (EZ) band. **2b**: A representation of a large cyst exhibiting lateral liquid dispersion (denoted by horizontal arrows), which subsequently instigates plateau development (highlighted by vertical arrows), followed by escalating compression on the outer retinal layer (ORL). **2c**: Portrayal of the cyst post-treatment, where resolution has been achieved, albeit with disruptions to the EZ, manifested as a discontinuous EZ (indicated by a dashed EZ band) **Legends**: RNFL, retinal nerve fiber layer; GCL, ganglion cell layer; IPL, inner plexiform layer; INL, inner nuclear layer; OPL, outer plexiform layer; ONL, outer nuclear layer; ELM, external limiting membrane; EZ, ellipsoid zone; IDZ, interdigitation zone; and RPE, retinal pigment epithelium

Only one eye of each type 2 diabetic patient, with or without DR, was selected for the study. When only one eye met the inclusion criteria, it was automatically selected. When both eyes were eligible, the study eye was selected via a random number generator program. Patients were subsequently segregated into three groups: patients without DME (group 1), patients with DME but without any cyst featuring a plateau in the lower region (group 2), and patients with cyst featuring an inferior cyst plateau (group 3). The cyst plateau was characterized as a flat conformation at its posterior aspect, which was manually measured from both edges via a device caliper tool (Fig. 1). To qualify for Group 3, the eyes had to present at least one cyst with a plateau within the inner Early Treatment Diabetic Retinopathy Study (ETDRS) ring. If a patient had more than one cyst with a plateau within the inner ETDRS ring, the measurements were recorded from the one with the greatest height.

### Systemic evaluation

We assessed medical records for demographic and medical data, including age, sex, race, and details about diabetes and other systemic diseases, such as systemic arterial hypertension (SAH). We also evaluated medical records for surgical and medical history and blood exams, such as blood tests, to determine fasting plasma glucose (FPG), glycosylated hemoglobin (A1C), and levels of serum creatinine and urea.

### Ocular clinical examination

A comprehensive ophthalmologic evaluation was performed on all participants, including clinical review, BCVA determination using a Snellen chart from a 4-meter distance (later converted to logMAR), slit-lamp examination, Goldmann applanation tonometry, and biomicroscopic retinal examination with a 78-diopter lens. All participants subsequently underwent SD-OCT (Spectralis, Heidelberg Engineering, Heidelberg, Germany). The severity of DR was classified during fundus examination as mild, moderate, or severe nonproliferative diabetic retinopathy (NPDR) or proliferative diabetic retinopathy (PDR) based on the International Clinical Disease Severity Scale [13]. 

### OCT analysis of the Retina

A Spectralis SD-OCT system equipped with eye-tracking dual-beam technology (Heidelberg Engineering GmbH, Heidelberg, Germany) was used. We analyzed the images with the Heidelberg Eye Explorer software (version 1.8.6.0) employing the HRA/Spectralis Viewing Module (version 5.8.3.0) with an axial resolution of approximately 5–7 μm. The macular volume scan encompassed a 20° × 20° field that was 6 × 6 mm, captured through 49 horizontal B-scans with an interscan distance of 120 μm.

Central macular thickness (CMT) was derived from a 1000-µm diameter ETDRS inner circle grid map placed over the macula with the assistance of built-in automated software that measures the distance between the internal limiting membrane (ILM) and the outer boundary of the retinal pigment epithelium (RPE). We diagnosed DME in cases where eyes presented intraretinal cysts and/or a CMT value exceeding 305 μm, grouping them accordingly [14]. 

A horizontal line scan crossing the foveal center was selected for the analysis of group 1 patients. The scan chosen for measuring the retinal parameters in patients with DME (groups 2 and 3) was the one where the cyst exhibited the greatest height within the first ETDRS circular grid with a diameter of 1 mm. A cyst was defined as a circular or ovoid intraretinal hyporeflective space present either in the inner nuclear layer (INL) or outer nuclear layer (ONL) devoid of lumen (Fig. 1). Using the caliper tool from Heidelberg Eye Explorer, we measured the following parameters: (1) Cyst height: the vertical distance between the upper and lower edges of the cyst; (2) Cyst plateau: a region with flat conformation at the posterior side of the cyst, whose length was determined as the horizontal distance between its edges (Fig. 1.2 b); (3) Outer retinal layer thickness (ORLt): the distance between the inner boundary of the external limiting membrane (ELM) and posterior boundary of the RPE beneath the center of the cyst and 500 μm temporally and nasally. For patients without a cyst (group 1), this measurement was performed in the foveal center; (4) Retina height: the distance between the ILM and the outer edge of the RPE; and (5) Disruption of the ellipsoid zone (EZ): noted when there was a focal discontinuity or impairment of the EZ band observable in the SD-OCT (Fig. 1.2c).

### Statistical analysis

All analyses were conducted with the IBM SPSS Statistics software, v. 15.0 (SPSS Inc., Chicago, IL. USA) and Stata 11.0. Initially, descriptive statistics were obtained for all variables under consideration. Continuous variables were characterized via mean and standard deviation (SD) values, whereas categorical variables were described via frequencies and percentages.

To assess the normality of the distribution, Shapiro‒Wilk’s W test complemented by graphical analysis was utilized. Comparative analyses were performed via the Mann–Whitney U test for quantitative variables between groups, whereas the chi-square test and McNemar test were employed for categorical variables. The Kruskal‒Wallis test was used to analyze nonparametric data.

The intergrader reliability for both quantitative and categorical variables was evaluated via the intraclass correlation coefficient for quantitative variables and Cohen’s kappa coefficient for categorical variables. An analysis of variance (ANOVA) was conducted to examine variations in means among groups, considering both quantitative and categorical descriptive variables in relation to cyst and ORL parameters. Subsequently, Scheffé’s test was employed to identify specific pairs exhibiting significant mean differences.

Additionally, a post hoc analysis was conducted via the Tukey honest significant difference (HSD) test to validate statistically significant comparisons. Correlational analyses examining cyst height, plateau, and ORLt in relation to both quantitative and qualitative variables were conducted via the Spearman correlation coefficient. The associated p values were adjusted via the Bonferroni correction.

Univariate and multivariate logistic and linear regressions were applied to scrutinize the associations of the cyst parameters with the categorical and quantitative variables of interest, respectively.

A p value less than 0.05 was considered statistically significant unless otherwise stated.

## Results

A total of 164 eyes were analyzed in this study. The participants were categorized into three groups: group 1 included 48 individuals (29.3%), group 2 comprised 47 individuals (28.7%), and group 3 consisted of 69 individuals (42%). The characteristics of the eyes examined in the study are outlined in Table 1.

**Table 1:** Baseline characteristics of control subjects and diabetic patients

Variables	Group 1	Group 2	Group 3	*p* value
*N* ** (patients) (%)**	48 (29.3)	47 (28.7)	69 (42.1)	< 0.0001
Age (years), mean (SD)	63 (7.8)	65 (8.4)	70 (5.7)	0.67
Sex: men. *N* (%)	25 (52.1)	23 (48.9)	38 (55.1)	0.87
Pseudophakic. *N* (%)	12 (25)	19 (40.4)	38 (55.1)	0.005
**DM type**				
Type 2. *N* (%)	48 (100)	47 (100)	69 (100)	
Insulin use. *N* (%)	19 (39.6)	7 (14.9)	28 (40.6)	0.008
A1C (%), mean (SD)	8.2 (1.95)	8.5 (1.65)	8.7 (1.48)	0.279
DM, mean duration (years) (SD)	6 (4)	7 (3)	11 (3)	< 0.0001
Presence of SAH in DM patients. *N* (%)	18 (37.5)	15 (31.9)	35 (50.7)	> 0.05
Serum creatinine, mean (SD)	0.90 (0.2)	1.1 (0.35)*	1.1 (0.36)*	0.003
Vision loss duration, mean (years) (SD)	0.25 (0.4)	2.62 (1.5)	4.4 (2.1)	< 0.0001
**Ophthalmic Measurements**				
Visual acuity (LogMAR), mean (SD)	0.02 (0.04)	0.15 (0.11)	0.45 (0.21)	< 0.0001
Central macular thickness (µm), mean (SD)	281.5 (15.8)*	324.2 (43.6)*	479.2 (106.9)*	< 0.001
**DR classification. ** *N* ** (%)**				
DM without DR	22 (45.8)	0	0	
Mild NPDR	15 (31.3)	2 (4.2)	0	
Moderate NPDR	11 (22.9)	25 (53.2)	16 (23.2)	
Severe NPDR	0	14 (29.8)	36 (52.2)	
PDR	0	6 (12.8)	17 (24.6)	

**Legends**: *p < 0.05; A1C, glycated hemoglobin; DM, diabetes mellitus; SAH, systemic arterial hypertension; DR, diabetic retinopathy; NPDR, nonproliferative diabetic retinopathy; PDR, proliferative diabetic retinopathy

In terms of BCVA, a statistically significant difference was observed between Group 3 and Groups 1 and 2 (*p* < 0.0001), whereas no notable difference was detected between Groups 1 and 2 (*p* > 0.05) (Table 1). The CMT displayed statistically significant differences across all three groups, as presented in Table 1. Moreover, a significant correlation between CMT and BCVA was identified (*r* = 0.36, *p* < 0.001). Further correlation analyses between retinal parameters and clinical data revealed a considerable influence of several variables on retinal characteristics. These relationships are summarized in Table 2.

**Table 2:** Correlations between demographic data and retinal parameters

		Cyst height	Cyst plateau	ORL thickness	CMT
**Age (years)**	r	0.150	-0.022	-0.147	0.259*
	p	0.110	0.857	0.060	0.001
**Pseudophakic**	r	-0.002	-0.002	-0.093	0.053
	p	0.980	0.984	0.237	0.499
**DM duration (years)**	r	0.365*	0.078	-0.256*	0.482*
	p	< 0.001	0.522	< 0.001	< 0.001
**DR stages**	r	0.422*	0.193	-0.346*	0.600*
	p	< 0.001	0.112	< 0.001	< 0.001
**Time of vision loss (years)**	r	0.558*	0.375*	-0.506*	0.645*
	p	< 0.001	0.001	< 0.001	< 0.001
**Insuline use**	r	0.367*	0.177	-0.220*	0.187†
	p	< 0.001	0.146	0.005	0.017
**SAH**	r	0.209†	-0.021	-0.147	0.183†
	p	0.024	0.864	0.061	0.019
**A1C**	r	0.138	0.275†	-0.184†	0.096
	p	0.140	0.022	0.018	0.223
**Creatinine**	r	0.013	-0.291†	0.022	-0.082
	p	0.893	0.015	0.782	0.297

**Legends**: * Correlation is significant at the *p* = 0.01 level. † Correlation is significant at the 0.05 level. CMT, central macular thickness. ORL, outer retinal layer. DM, diabetes mellitus. DR, diabetic retinopathy. SAH, systemic arterial hypertension

The intraclass correlation coefficients for various parameters, including the ORLt beneath the cyst, cyst height and the cyst plateau length, were recorded as 0.98, 0.96, and 0.97, respectively. Cohen’s kappa coefficient for the EZ disruption was 0.85.

The dimension of cyst height (mean ± SD, in µm) was significantly different between groups 2 (87 ± 57) and 3 (299 ± 169), with a p value < 0.0001. The mean ± SD cyst plateau length of the eyes in group 3 was 493 ± 436 μm. Both the cyst height and the cyst plateau length were significantly positively correlated with BCVA, with correlation coefficients of *r* = 0.58 and *r* = 0.64, respectively (*p* < 0.0001).

In analyzing the difference in cyst height according to groups 2 and 3 based on the presence of the plateau, the area under the curve yielded favorable results (AUC = 0.921). The optimal cutoff point for cyst height that best discriminates plateau formation was determined to be 130.5 μm, with a sensitivity of 89.9% and specificity of 83%.

Further analyses, outlined in Table 3, demonstrated the ORLt at specific locations relative to the cyst across all groups. Notably, the ORL beneath the cyst was significantly thinner in patients in group 3 than in those in groups 1 and 2 (*p* < 0.0001). However, no significant difference in this parameter was observed between groups 1 and 2 (*p* = 0.73). Additionally, ORLt was significantly negatively correlated with BCVA (*r* = − 0.48, *p* < 0.0001).

**Table 3:** Mean (SD) outer retinal layer thickness

	500 μm nasal	Beneath cyst	500 μm temporal	*p*
**G1 (no cyst)**	85 (6)	84 (7)*	84 (6)	> 0.05
**G2 (cyst without plateau)**	85 (7)	82 (8)	84 (6)	> 0.05
**G3 (cyst with plateau)**	81 (17)	71 (15)^†^	78 (14)	< 0.001

**Legends**: * For group 1, the ORLt measurement corresponding to the point beneath the center of the cyst was performed in the foveal center. ^†^*p* = 0.0001 between groups 1 and 2, measured beneath the cyst. Group 1, diabetic patients without cysts; Group 2, diabetic patients with cysts without plateaus; Group 3, diabetic patients with cysts and plateaus

In groups 2 and 3, which included only patients with cysts, none of the patients in group 2 presented with EZ disruption, in contrast to 34 individuals (49.3%) in group 3. This difference was statistically significant (*p* < 0.0001). The presence of EZ disruption was also associated with the dimensions of the cyst height, the cyst plateau and the ORLt beneath the cyst (*p* < 0.0001, as determined through the Mann‒Whitney U test). Moreover, EZ disruption was significantly associated with BCVA (*r* = 0.55, *p* < 0.0001).

**Fig. 2:**
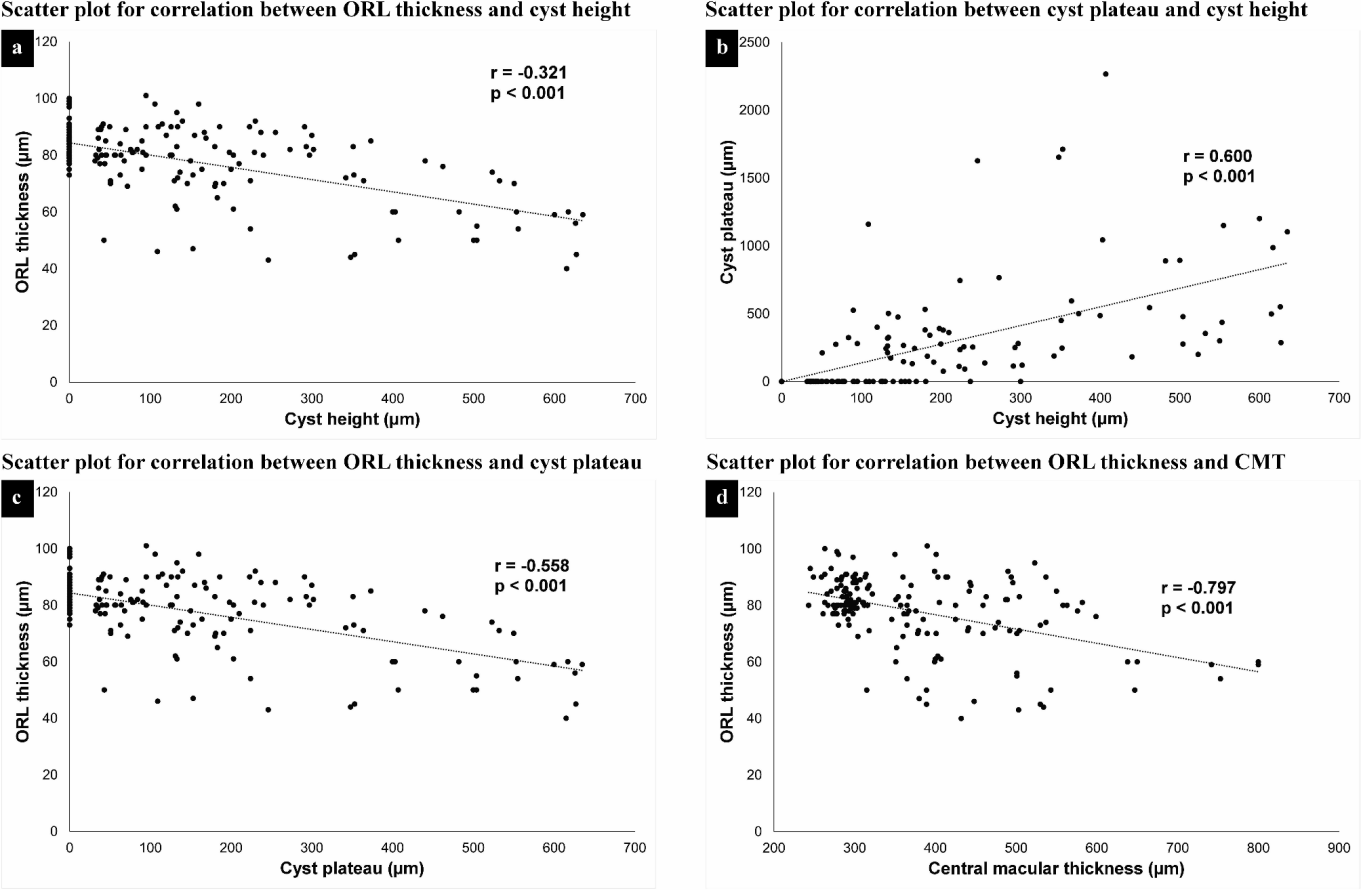
**a** and **b**: Scatter plots illustrating the direct correlation between cyst height and outer retinal layer thickness (ORL) and between cyst height and cyst plateau length, respectively. c and d: Scatter plots demonstrating the inverse correlation between the cyst plateau and ORL thickness and between central macular thickness and ORL thickness, respectively

Significant correlations were identified among various retinal parameters. The cyst height dimension was negatively correlated with ORLt beneath the cyst (*r* = − 0.32, *p* < 0.001) and positively correlated with the length of the cyst plateau (*r* = 0.60, *p* < 0.001). The ORLt beneath the cyst was negatively correlated with the cyst plateau length (*r* = − 0.56, *p* < 0.001) and central macular thickness (*r* = − 0.8, *p* < 0.001) (Fig. 2).

For every increase of 10 μm in the plateau width, there is a reduction of 0.16 μm in ORLt. Additionally, an increase in the cyst height of 10 μm results in a reduction of 0.29 μm in ORLt, independent of the other parameters.

The multivariate linear regression revealed that, among the analyzed retinal parameters, only the cyst plateau was significantly associated with BCVA (*p* < 0.001). However, no statistically significant correlation was observed between cyst height or ORLt and BCVA (*p* = 0.14 and *p* = 0.07, respectively). Furthermore, a multivariate logistic regression model revealed that none of the following investigated retinal parameters was associated with EZ disruption: cyst height (*p* = 1.0), cyst plateau (*p* = 0.09), ORLt (*p* = 0.50) or CMT (*p* = 0.50).

## Discussion

This study aims to enhance our understanding of the complex mechanisms underlying visual impairment in DME patients, investigating large cysts as a potential biomarker for visual deterioration in DME patients.

**Fig. 3:**
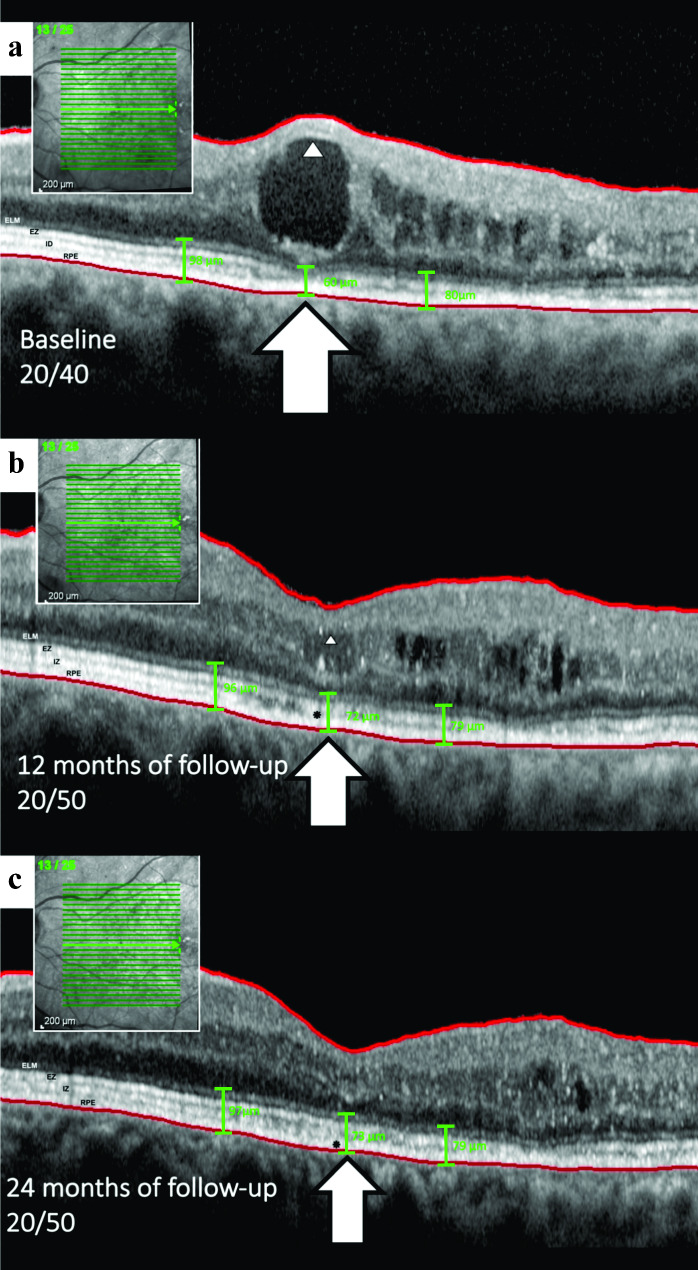
Sequential evolution of a cyst in the inner nuclear layer (INL) and subsequent impingement on the outer retinal layer (ORL) in an included patient. **a**, Following the formation and growth phase, large cysts begin to compress the inner layers, as indicated by the arrowhead, which exhibits hyperreflectivity due to the compaction of cells and impinges on the ORL, as shown by the arrow. In this instance, the external limiting membrane (ELM), ellipsoid zone (EZ), and interdigitation zone (IZ) are thinned and appear to disappear. The ORL thickness measured at different points - underneath the cyst and at 500 microns temporal and nasal - were 60, 80, and 98 microns, respectively. **b**, Over the one-year follow-up under bevacizumab treatment, the cyst height decreased, allowing the ellipsoid zone to be identifiable once more, albeit with disruption (indicated by a black asterisk). Concurrent ORL thinning is also observed. The ORL thickness was measured at different points: 72, 79, and 96 microns underneath the cyst and 500 microns both temporally and nasally. **c**, Ultimately, there is noticeable disruption of the ellipsoid zone and ORL thinning after the resolution of the cyst, without concurrent retinal pigment epithelium (RPE) atrophy in this case, as marked by the black asterisk

In the present study, we identified a correlation between large central macular intraretinal cysts and worse BCVA outcomes compared to their smaller counterparts, corroborating earlier studies [7]. We also observed a moderate correlation between CMT and cyst height with BCVA, which is consistent with earlier publications [3, 4, 9, 15–17]. Existing studies, including post hoc analyses of the RIDE/RISE and RESTORE/RESTORE-extension studies, support the hypothesis that an increase in cyst size may lead to poorer visual outcomes [18]. Large foveal cysts, mainly those greater than 380 μm, exhibit a significant deterioration in BCVA even after post-treatment reduction [4]. Figure 3 illustrates a case that supports the aforementioned statement and aligns with the results of our study.

When evaluating the CMT, we observed that it is composed of cysts of various sizes. Smaller intraretinal cysts located at the foveal center have been associated with better visual outcomes compared to larger cysts, as noted by Gerendas and Pelosini et al. [4, 9] Thus, when CMT is predominantly composed of smaller cysts, visual function tends to be less affected than when larger cysts are present. This variability in cyst size may help explain the low to moderate correlation between CMT and BCVA reported in multiple studies. Our findings suggest that there may be a threshold in cyst growth, beyond which retinal neurons are at greater risk of damage. This emphasizes the critical need for timely intervention in the management of DME and highlights the importance of early treatment to preserve visual function.

We also explored the evolving morphology of enlarging cysts, whereupon reaching a critical volume – constrained by the retina’s accommodative elasticity – they begin to spread laterally, changing their characteristic spherical form to a posterior flat conformation, i.e., the cyst plateau. This transformation may be influenced by the relative stiffness of the ORL, leading to photoreceptor compression. Indeed, when evaluating the general trend of the degree of elasticity across the various retinal layers, a gradual increase in rigidity is observed towards the outermost layers, with the photoreceptor layer being the least elastic [19, 20]. This phenomenon could explain the significant correlation observed in our study between the cyst plateau and worse BCVA, as well as a higher incidence of EZ disruption.

In a groundbreaking study, Karahan et al. reported that cysts within the retina can exert substantial pressure on the internal retinal layers, potentially leading to significant damage. They developed a mathematical formula that demonstrated that, in some scenarios, the cyst could generate a pressure of approximately 60 mmHg, potentially impeding axoplasmic flow and causing injury to the retinal nerve fiber layers [10]. However, their model proposed that the pressure exerted by the intraretinal cystoid space was equivalent to the force applied per unit area, a simplification that may not fully capture the complexity of the process involved. Although our study did not investigate the effects of cysts on the inner retinal layers, we propose an additional explanation through the mechanical compression of cysts on the ORL to rationalize the more deleterious impact on vision observed with large cysts compared to smaller ones.

**Fig. 4:**
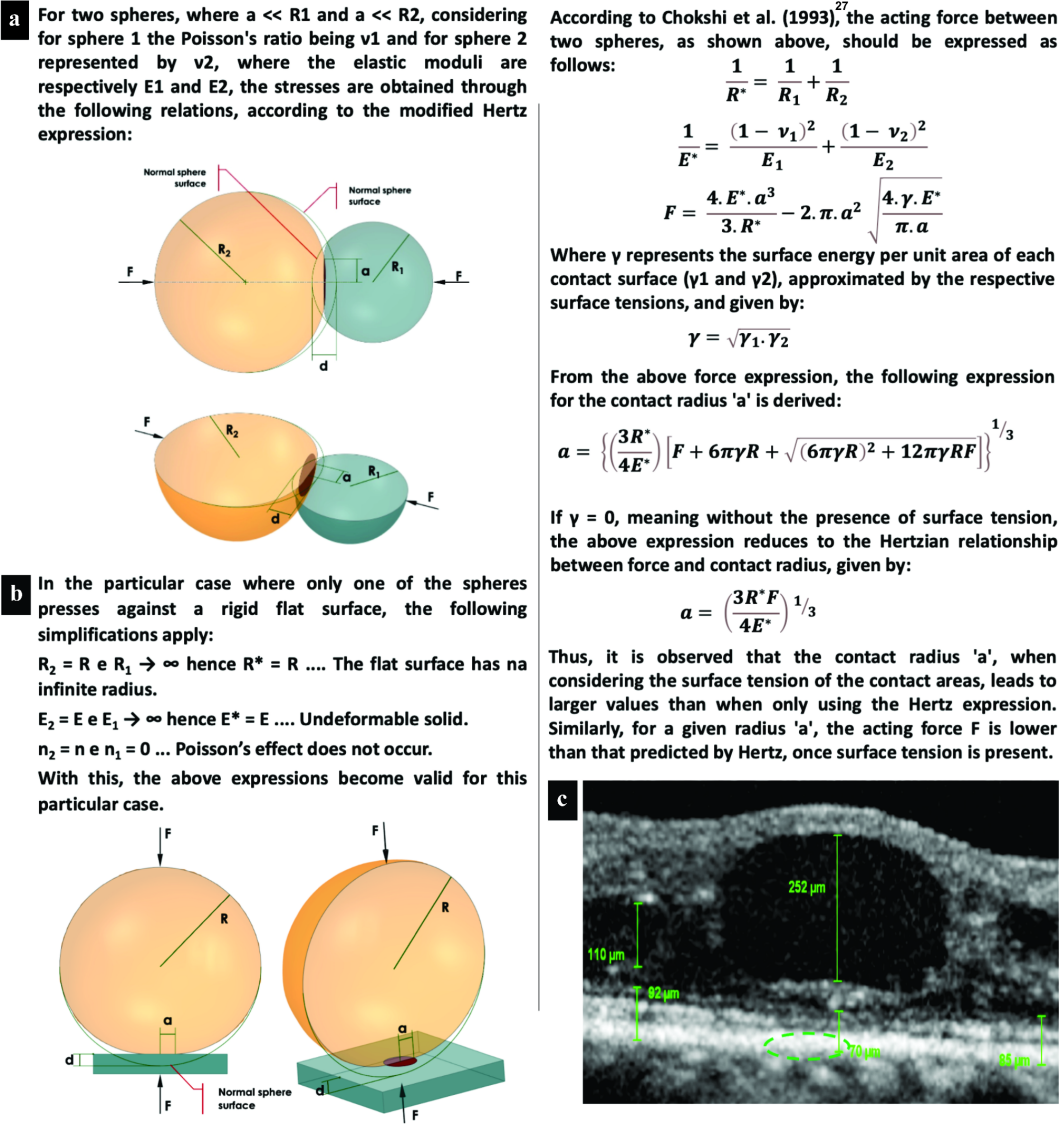
Schematic representation of contact stress and deformation according to contact mechanics. **a**, The image illustrates two interacting spheres where the point of maximum contact pressure is located centrally, resulting in a semi elliptic pressure distribution, as depicted by the green dashed elliptic area in C. **b** and **c**, These images demonstrate that the theory is applicable even when a spheroid object, such as a retinal cyst, engages with a more or less flat surface, akin to the retina. This principle holds because even a “flat” plate can be equated to a sphere with an infinite radius, as shown in B **Legends**: F, forces between the bodies; R, radii of curvature; a, radius of the contact area; d, depth of deformation; E, moduli of elasticity; ν, Poisson’s ratios; d, depth of identation; R*, flat surface with an infinite radius; E*, undeformable solid

To further explore this phenomenon, we suggest applying Hertz’s theory of contact mechanics [12]. This theory can be used to measure the tension in the contact area, depending on the applied forces, the radii of curvature of the two bodies, and their moduli of elasticity. It is also possible to calculate the size of the contact area and the depth of deformation of the two bodies, represented by simple geometric figures, such as two spheres or a sphere and a flat surface (Fig. 4a and b).

According to Hertz´s theory, the maximum contact pressure arises at the center of the interacting spheres, giving rise to a semi elliptic pressure distribution. Theoretically, the contact area of two spheres is a single point. As a result, high pressure, which tends to infinity, arises between the two curved surfaces, causing immediate deformation of both surfaces. However, owing to the elastic deformation of the bodies, the contact point is converted into a small contact area [12, 21]. We believe that this theory can be useful in understanding the development of a posterior flat conformation in large cysts when certain critical volumes are reached according to the accommodative elasticity of the retina. Nevertheless, the classical Hertz model has limitations since it remains accurate only when the ratio of the indentation depth to the indenter radius is less than 0.1 [22]. 

In real-world scenarios, materials with different properties interact, leading to a diverse range of indentations. This has spurred several authors to evolve the Hertz model to characterize contact behaviors under finite indentations using both linear and nonlinear regimes of elasticity [23–27], akin to the approach we have adopted in our study. These refined models (modified theories), validated through comparative simulations with the classical Hertz theory, revealed congruent curve patterns, thereby substantiating the applicability of the fundamental principles of the theory.

Furthermore, when applying the modified Hertz theory in scenarios involving liquids, it is imperative to account for surface tension — a critical factor highlighted in Chokshi’s 1993 publication. The article ingeniously incorporated this aspect into Hertzian theory, underscoring its practicality in elucidating the microphysics of coagulation between two colliding, smooth, spherical grains at the elastic limit and demonstrating its adaptability to analyses involving diverse materials or tissues [28]. 

In our clinical observations, we noted that large cysts exhibit signs of compressing the ORL, resulting in its thinning (Fig. 4c) and adopting a flat punch shape of pressure distribution [29]. This phenomenon can be conceptualized through Hertzian theory, which outlines how perpendicular and tangential forces interact between spheres at the point of contact (Fig. 4a). Intriguingly, this theory retains its applicability even when a spherical entity, such as a retinal cyst, contacts a substantially flat surface, such as the ORL (Fig. 4c). This is attributable to the theoretical equivalence of a flat plate to a sphere with an infinite radius (Fig. 4b).

In alignment with the assumption that a retinal cyst functions analogously to a sphere, our investigation suggests the presence of both perpendicular and tangential contact forces at the junction between the cyst and the ORL. Furthermore, as the cyst enlarges, increasing its contact with the ORL, reciprocal deformation occurs, characterized by plateauing and compression phenomena, respectively (Fig. 1.2b, Fig. 3a, and Fig. 4c) [11]. 

In our cohort, ORL thinning was observed exclusively in eyes with a plateau, and this thinning was correlated with both worse BCVA and increased cyst height according to multivariate regression analysis. Despite the absence of a direct correlation between ORLt and BCVA, a greater number of eyes with ORL thinning exhibited a decline in BCVA. We hypothesize that the minor variations in ORLt, combined with the sample size, might be the underlying factors preventing the achievement of statistical significance.

In light of the findings gained from this research, we acknowledge the suitability of the modified Hertz’s theory of contact mechanics as a congruent model to elucidate our observations. This theoretical framework provides a more profound understanding of the adverse effects that large intraretinal cysts exert on the retina and vision, thereby reinforcing its crucial role as a pivotal biomarker in diabetic macular edema (DME).

Our findings, while clinically significant, should be approached with caution due to several limitations inherent to this study. There is potential for other underlying structural alterations induced by DR concurrent with macular edema; these alterations can affect the integrity of both the ORL and the inner retinal layers, leading to damage to other cells, including bipolar cells, thereby contributing to visual dysfunction. Moreover, the relatively small sample size constitutes a limitation, as the compressed inner retinal layers situated above the cyst were not evaluated for thickness, nor was the foveal avascular zone measured. Although linear rather than volumetric measurements were taken during the analysis of the morphological characteristics of the cysts, the results obtained provide practicality for clinical use. Given these constraints, we recommend interpreting the results carefully while acknowledging the need for further research to substantiate our findings with a more comprehensive analysis encompassing a larger and more diverse patient cohort.

## Conclusions

Our study suggests that large central intraretinal cysts in DME, while causing disruptions in the synapses within the inner retinal layers between ganglion cells and photoreceptors, also induce compression and deformation of the ORL, leading to photoreceptor impairment and eventual visual degradation. For this reason, large cysts can assume a flat configuration in their inferior region, termed a plateau, which signifies compression of the ORL and is associated with morphological damage to the outer retina.

These findings can be understood based on the modified Hertz’s theory of contact mechanics, as large cysts can compress the outer retina, damaging the photoreceptors. Consequently, we advocate for sustained exploration and scrutiny of retinal cysts through the laws of contact mechanics, a scientific perspective poised to add significant value to the nuanced understanding and management of this condition.

## Data Availability

The datasets generated and/or analyzed during the current study are not publicly available owing to participant confidentiality and privacy concerns but are available upon reasonable written request. The requested data are shared following a signed data access agreement. Data requests can be made by contacting the corresponding author (micael_valtoni@hotmail.com). The data will be made available for two years from publication.

## Abbreviations

A1C: Glycosylated hemoglobin
BCVA: Best-corrected visual acuity
CMT: Central macular thickness
DME: Diabetic macular edema
DR: Diabetic retinopathy
DRIL: Disorganization of retinal inner layers
ELM: External limiting membrane
ETDRS: Early treatment diabetic retinopathy study
EZ: Ellipsoid zone
FPG: Fasting plasma glucose
ILM: Internal limiting membrane
INL: Inner nuclear layer
IZ: Interdigitation zone
NPDR: Nonproliferative diabetic retinopathy
OCT: Optical coherence tomography
ONL: Outer nuclear layer
ORL: Outer retinal layer
ORLt: Outer retinal layer thickness
PDR: Proliferative diabetic retinopathy
RPE: Retinal pigment epithelium
SAH: Systemic arterial hypertension
SD-OCT: Spectral-domain optical coherence tomography
